# Apatinib Inhibits Cell Proliferation and Induces Autophagy in Human Papillary Thyroid Carcinoma via the PI3K/Akt/mTOR Signaling Pathway

**DOI:** 10.3389/fonc.2020.00217

**Published:** 2020-03-11

**Authors:** Xiangrui Meng, Huijuan Wang, Jingzhu Zhao, Linfei Hu, Jingtai Zhi, Songfeng Wei, Xianhui Ruan, Xiukun Hou, Dapeng Li, Jun Zhang, Weiwei Yang, Biyun Qian, Yu Wu, Yuan Zhang, Zhaowei Meng, Lizhao Guan, Huilai Zhang, Xiangqian Zheng, Ming Gao

**Affiliations:** ^1^Key Laboratory of Cancer Prevention and Therapy, Department of Lymphoma, National Clinical Research Center for Cancer, Tianjin's Clinical Research Center for Cancer, Tianjin Medical University Cancer Institute and Hospital, Tianjin, China; ^2^Key Laboratory of Cancer Prevention and Therapy, Department of Thyroid and Neck Tumor, National Clinical Research Center for Cancer, Tianjin's Clinical Research Center for Cancer, Tianjin Medical University Cancer Institute and Hospital, Tianjin, China; ^3^Department of Otolaryngology-Head and Neck Surgery, Tianjin First Center Hospital, Tianjin, China; ^4^Department of Epidemiology, School of Public Health, Shanghai Jiao Tong University, Shanghai, China; ^5^Department of Head and Neck Surgery, Fujian Cancer Hospital, Fujian Medical University Cancer Hospital, Fujian, China; ^6^Department of Head and Neck Surgery, Jiangsu Cancer Hospital, Jiangsu Institute of Cancer Research, Nanjing Medical University Affiliated Cancer Hospital, Nanjing, China; ^7^Department of Nuclear Medicine, Tianjin Medical University General Hospital, Tianjin, China; ^8^Department of Biochemistry and Molecular Biology, School of Basic Medical Sciences, Tianjin Medical University, Tianjin, China

**Keywords:** apatinib, papillary thyroid carcinoma, PI3K, apoptosis, autophagy

## Abstract

**Background:** Patients with metastatic radioiodine-refractory papillary thyroid carcinoma (PTC) have limited treatment options and a poor prognosis. There is an urgent need to develop new drugs targeting PTC for clinical application. Apatinib, a novel small-molecule tyrosine kinase inhibitor (TKI), is highly selective for vascular endothelial growth factor receptor-2 (VEGFR2) and exhibits antitumor effects in a variety of solid tumors. Although apatinib has been shown to be safe and efficacious in radioiodine-refractory differentiated thyroid cancer, the mechanism underlying its antitumor effect is unclear. In this report, we explored the effects of apatinib on PTC *in vitro* and *in vivo*.

**Methods:** VEGFR2 expression levels were evaluated by immunohistochemistry (IHC), qPCR, and western blotting (WB). The effects of apatinib on cell viability, colony formation, and migration in the Transwell assay were assessed *in vitro*, and its effect on tumor growth rate was assessed *in vivo*. In addition, the levels of proteins in signaling pathways were determined by WB. Finally, the autophagy level was assessed by WB, immunofluorescence (IF), and transmission electron microscopy.

**Results:** We found that high VEGFR2 expression is associated with tumor size, T stage, and lymph node metastasis in patients with PTC and that apatinib inhibits PTC cell growth, promotes apoptosis, and induces cell cycle arrest through the PI3K/Akt/mTOR signaling pathway. Moreover, apatinib induces autophagy, and pharmacological inhibition of autophagy or small interfering RNA (siRNA)-mediated targeting of autophagy-associated gene 5 (ATG5) can further increase PTC cell apoptosis.

**Conclusion:** Our data suggest that apatinib can induce apoptosis and autophagy via the PI3K/Akt/mTOR signaling pathway for the treatment of PTC and that autophagy is a potential novel target for future therapy in resistant PTC.

## Introduction

Thyroid cancer is the most common endocrine system tumor, and 53,990 new cases were predicted in 2018 (1). Standard treatments for papillary thyroid carcinoma (PTC), the most common type of thyroid cancer, include surgery, thyroid-stimulating hormone suppression, and radioiodine treatment. With these standard treatments, most patients have a good prognosis; however, 5% of patients present with radioiodine-refractory thyroid cancer and distant metastasis (2, 3). Currently, few treatment options are available for these patients. Therefore, new therapeutic strategies are urgently needed for PTC patients with advanced disease.

With an improved understanding of the molecular mechanisms of thyroid cancer, several important tumorigenic factors have been identified as new targets for antitumor therapy (4–6). A series of antitumor drugs, especially multiple targeted tyrosine kinase inhibitors (TKIs), have been developed for clinical treatment. Among them, sorafenib and vandetanib have been approved as standard therapies for advanced PTC (7, 8). In addition, an increasing number of TKIs are undergoing clinical trials. Apatinib is a new oral TKI that inhibits vascular endothelial growth factor receptor-2 (VEGFR2) with high selectivity. Apatinib has been approved as a safe and effective drug for patients with advanced gastric cancer for whom standard chemotherapy has failed (9, 10). In addition, apatinib has shown antitumor effects in various types of solid tumors in a number of ongoing phase II and III clinical trials (11, 12). Lin et al. (13) reported that apatinib showed promising efficacy in a small number of patients with radioiodine-refractory differentiated thyroid cancer. However, the antitumor mechanism of apatinib in PTC is unclear.

Drug resistance, a perpetual theme in the field of cancer treatment, is associated with multiple mechanisms. A series of studies have shown that autophagy activity plays an important role in TKI resistance. Autophagy is a catabolic process that is prevalent in eukaryotic cells that provides energy to support metabolism and survival by degrading proteins and organelles and maintains cell homeostasis (14). Accumulating evidence associates autophagy with adaptive drug resistance in multiple tumors. Patients with chronic lymphocytic leukemia who presented a higher level of autophagy were reported to exhibit a shorter survival time after treatment with imatinib than those who presented a lower level of autophagy, and imatinib treatment was more efficacious in patients with a higher level of autophagy. The same phenomenon was observed in the treatment of differentiated thyroid cancer with vemurafenib (15). Vemurafenib, a selective RAF inhibitor, did not achieve good results in the treatment of B-Raf mutant thyroid cancer, and the mechanism of drug resistance was shown to be related to autophagy induced by vemurafenib. Therefore, autophagy inhibition has become a potential target for TKI-resistant cancers.

In this study, we found that apatinib had an antitumor effect on PTC *in vitro* and *in vivo*. We also discovered that apatinib could induce protective autophagy. Notably, we investigated the relationship between apoptosis and autophagy induced by apatinib and inferred that targeting apatinib-induced autophagy has potential therapeutic benefit in the treatment of PTC.

## Materials and Methods

### Reagents

Apatinib was kindly provided by Jiangsu Hengrui Medicine Company (Jiangsu, China). The following primary antibodies were used: anti-VEGFR2, anti-Akt, anti-phospho-Akt (anti-p-Akt), anti-mTOR, anti-phospho-mTOR (anti-p-mTOR), anti-P70S6K, anti-phospho-P70S6K (anti-p-P70S6K), anti-ULK1, anti-phospho-ULK1 [anti-p-ULK1 (Ser 757)], anti-cyclin D1, anti-Bcl-2, anti-cleaved caspase3, anti-P21, anti-Bax, anti-ATG5, anti-P62/SQSTM1, anti-LC3B, and anti-Ki-67 antibodies (Cell Signaling Technology, Danvers, MA, USA), and anti-GAPDH and anti-cleaved PARP antibodies (GeneTex, Irvine, CA, USA). Hydroxychloroquine (HCQ) and SC79 were purchased from Selleck Chemicals (Houston, TX, USA).

### Cell Lines and Cell Culture

Four PTC cell lines (BCPAP, K1, KTC-1, and TPC-1 cells), two ATC cell lines (CAL-62 and 8505C cells), and one normal follicular epithelial cell line (Nthy-oris-3 cells; N9) were used in this study. K-1, TPC-1, and N9 cells were purchased from American Type Culture Collection (ATCC, USA), and the other cell lines were purchased from the Type Culture Collection of the Chinese Academy of Sciences (Shanghai, China). All the cell lines used in our experiments were routinely authenticated with short tandem repeat DNA profiling analysis by us or the investigator from which we received the cells, and the passage number of the cells used for experiments was ~20–30 in our laboratory. The cells were cultured in RPMI-1640 or DMEM supplemented with 10% FBS and 1% antibiotics (penicillin and streptomycin). All the cells were maintained in a humidified incubator at 37°C with 5% CO_2_. All culture reagents were purchased from Gibco (Grand Island, NY, USA).

### Clinical Data and Tissue Samples

A total of 187 patients diagnosed with PTC between January 2013 and June 2013 at Tianjin Medical University Cancer were enrolled in the study. Paraffinized blocks of cancer tissue were used to generate tissue microarrays (TMAs), with a random selection of 43 adjacent normal thyroid follicular tissues used as a control. Fresh cancer and adjacent normal thyroid follicular tissue specimens were collected from 22 PTC patients. All patients provided informed consent. This study was performed with the approval of the Tianjin Medical University Institutional Review Board.

### Animals

Twenty 4-week-old female NSG mice weighing 20 to 22 g purchased from SPF Biotechnology (Beijing, China) were used for the K-1 cell xenografts. All mice were housed under pathogen-free conditions. All animal experiments were carried out with the approval of the Ethics Committee of the Tianjin Medical University Cancer Institute and Hospital.

### Immunohistochemistry

The 3 μm sections on the TMA and from the paraffin block were subjected to immunohistochemical staining for VEGFR2, cleaved caspase3, and Ki-67 according to standard immunohistochemistry (IHC) protocol. Signals were amplified using a DAB substrate kit. Images were taken with an Olympus BX51 microscope. The results were evaluated independently by two experienced pathologists and scored with the H-score method.

### RNA Extraction and Quantitative RT-PCR

Total RNA was extracted from tissues and cells by using a TRIzol reagent (Invitrogen, Carlsbad, CA, USA), and total RNA was reverse-transcribed to cDNA using PrimeScript RT Master Mix (TaKaRa, Tokyo, Japan). To quantify VEGFR2 and actin mRNA levels, quantitative RT-PCR was performed on the reverse-transcribed products with SYBR Premix Ex Taq II (TakaRa, Tokyo, Japan) and specific primers. The sequences of the primers were as follows: 5′-GTGATCGGAAATGACACTGGAG-3′ and 5′-CATGTTGGTCACTAACAGAAGCA-3′.

### Cell-Viability and Colony-Formation Assays

The cell counting kit-8 (CCK8, Dojindo, Japan) assay was used as previously described to assess cell viability. The IC50 was calculated from survival curves using GraphPad Prism 7.0. For the colony-formation assay, cells were seeded at 500 cells per well in 6-well plates on the day before the experiment and then cultured in a medium containing apatinib at different concentrations for 2 weeks. Cell colonies were stained with 0.1% crystal violet before being photographed.

### Cell Apoptosis and Cell Cycle Analyses

Cells were treated with apatinib at different concentrations for 24 h, collected by trypsinization, washed twice with PBS, and stained with staining solution from an Annexin V/FITC detection kit. The samples were analyzed by flow cytometry according to the kit's instructions.

Cells were treated with apatinib at different concentrations for 24 h, collected by trypsinization, and washed twice with PBS. The harvested cells were fixed with 70% cold ethanol at −20°C overnight and stained with a propidium iodide (PI) solution containing RNase. Samples were analyzed by flow cytometry.

### Protein Extraction and Western Blotting (WB)

Proteins were extracted from different cells by using protease and phosphatase inhibitors according to the manufacturer's instructions, and the protein concentration was quantified using the BCA method. Equal amounts of protein were resolved by 8%−12% SDS-PAGE and transferred to PVDF membranes. Primary antibodies against the following proteins were used: VEGFR2, Akt, p-Akt, mTOR, p-mTOR, P70S6K, p-P70S6K, ULK1, p-ULK1 (Ser 757), cleaved PARP, Bcl-2, Bax, cleaved caspase3, cyclin D1, P21, P62, ATG5, and LC3B.

### Immunofluorescence (IF) Staining

Cells were cultivated on coverslips, incubated with 5 μM apatinib for 24 h, fixed in 4% paraformaldehyde for 20 min, and then permeabilized with 0.2% Triton X-100 for 5 min. After being blocked with 5% BSA for 1 h, the cells were incubated with anti-LC3B primary antibody overnight at 4°C. Then, the cells were washed, incubated with secondary antibody, and stained with 4',6-diamidino-2-phenylindole (DAPI) (Solarbio, Beijing, China). Cells mounted on coverslips were observed by confocal microscopy.

Cells were incubated with apatinib with or without HCQ in the above steps.

### Transmission Electron Microscopy

The ultrastructure of autophagosomes in the treated cells was identified by transmission electron microscopy as described previously (16).

### RNA Interference

Small interfering RNA (siRNA) targeting ATG5 was designed and synthesized by GenePharma (Shanghai, China). K-1 cells were transfected with ATG5 siRNA with Lipofectamine 2000 according to the manufacturer's instructions. The total protein was extracted 48 h after transfection as described previously (17).

### Xenograft Model

All mice were housed under pathogen-free conditions. All animal experiments were carried out with the approval of the Ethics Committee of the Tianjin Medical University Cancer Institute and Hospital. K1 cells (5 × 10^6^ in 0.1 ml of serum-free medium/mouse) were injected subcutaneously into the right buttock of each mouse. When the tumor grew to 3 × 3 mm^2^, the mice were randomly assigned to four groups, and the five mice in each group were administered dimethyl sulfoxide (DMSO), apatinib (100 mg/kg/day orally), HCQ (60 mg/kg/day interperitoneally), or a combination of apatinib and HCQ for 28 days. The mice were then monitored for an additional 14 days. The tumors were measured every 4 days with digital calipers.

### Statistical Analysis

GraphPad Prism version 7.0 (La Jolla, CA, USA) was used for statistical analyses. Differences among variants were assessed by the chi-square test, and comparisons between groups were analyzed by one-way ANOVA. *P*-values indicating significant differences are shown in the figures as follows: ^***^p < 0.001, ^**^p < 0.01, ^*^p < 0.05.

## Results

### VEGFR2 Expression Was Elevated in PTC

**Figure 1:**
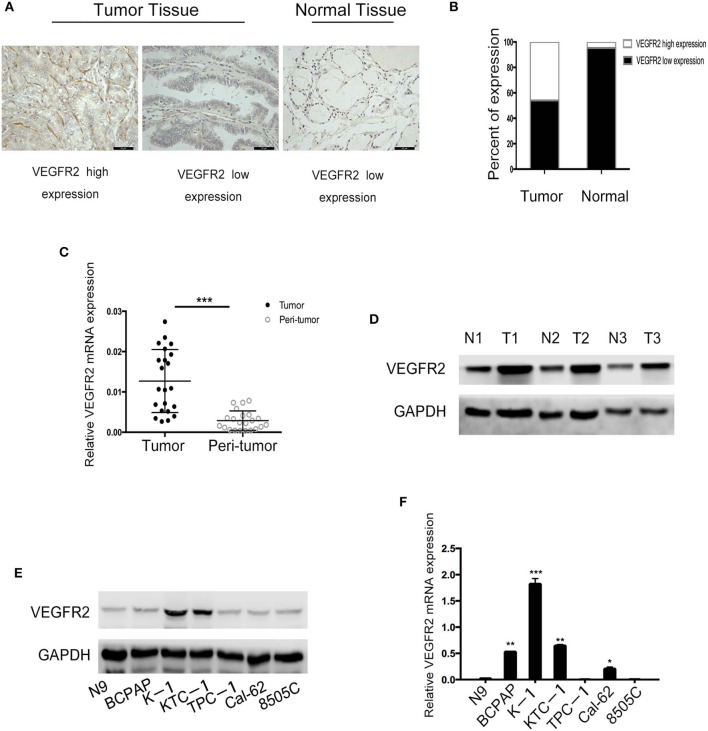
VEGFR2 expression is elevated in PTC. **(A)** Immunohistochemical staining of a TMA containing PTC and normal thyroid follicular tissue specimens for VEGFR2. **(B)** VEGFR2 expression in PTC and normal thyroid follicular tissue. **(C)** VEGFR2 mRNA levels in PTC and normal thyroid follicular tissue. **(D)** Western blot assay showing increased VEGFR2 expression in PTC tissue compared to normal thyroid tissue. **(E)** Western blot assay of VEGFR2 expression in thyroid cell lines. **(F)** VEGFR2 mRNA levels in thyroid cell lines. Data are expressed as the mean ± SD (*P < 0.05, **P < 0.01, ***P < 0.001 vs. N9 cells).

VEGFR2 expression was evaluated in 187 cases of PTC and 43 adjacent normal thyroid follicular epithelial tissues through IHC. The expression of VEGFR2 at the plasma membrane and in the cytoplasm was detected. We observed that most signals were detected from cancer cells (numerous true papillae and ground glass nuclei compared with follicular epithelial cells), as shown in Figure 1A. VEGFR2 expression was higher in thyroid cancer tissue than in normal thyroid follicular tissue (Figure 1B and Table 1). Meanwhile, a high level of VEGFR2 expression was associated with tumor size, T stage, lymph node metastasis, and tumor node metastasis (TNM) stage (Table 2). To further explore VEGFR2 expression in PTC, RT-PCR was used to detect VEGFR2 mRNA levels in fresh specimens from 22 PTC patients; these mRNA levels were obviously higher in PTC tissues than in normal thyroid follicular tissues (Figure 1C). Three of the 22 patients were randomly selected for an analysis of VEGFR2 protein expression in tissue by WB, the results of which showed that VEGFR2 expression was higher in cancer tissue than in normal tissue (Figure 1D). Next, we examined VEGFR2 protein and mRNA levels in seven thyroid cell lines, including normal thyroid follicular epithelial cells, PTC cell lines, and anaplastic thyroid cancer cell lines. VEGFR2 expression in the K-1 and KTC-1 PTC cell lines was higher than that in the other cell lines (Figures 1E,F). These data suggest that VEGFR2 expression is elevated in PTC.

**Table 1:** VEGFR2 expression in thyroid cancer and normal thyroid follicular tissue.

**VEGFR2 expression**	**Tumor tissue**	**Normal tissue**	***χ^2^***	***p*-value**
Low	101 (54%)	41 (95.4%)		
High	86 (46%)	2 (4.6%)	23.571	0.000

**Table 2:** Relationship between clinicopathological features and VEGFR2 expression in PTC (*n* = 187).

**Clinicopathological feature**	**VEGFR2 expression**		***χ^2^***	***p*-value**
	**Low**	**High**		
**Age**				
<55	81 (52.6%)	73 (47.4%)	0.702	0.402
≥55	20 (60.6%)	13 (39.4%)		
**Gender**				
Male	22 (41.5%)	31 (58.5%)	4.654	0.031
Female	79 (59.0%)	55 (41.0%)		
**Tumor size**				
>1	47 (40.2%)	70 (59.8%)	24.102	0.000
≤ 1	54 (77.1%)	16 (22.9%)		
**Multifocality**				
Absent	65 (50.8%)	63 (49.2%)	1.703	0.192
Present	36 (61.0%)	23 (39.0%)		
**T stage**				
I–II	98 (58.0%)	71 (42.0%)	11.182	0.001
III–IV	3 (16.7%)	15 (83.3%)		
**Lymph node metastasis**				
Absent	70 (76.9%)	21 (23.1%)	37.464	0.000
Present	31 (32.3%)	65 (67.7%)		
**TNM stage**				
I–II	98 (56.3%)	76 (43.6%)	5.382	0.02
III–IV	3 (23.1%)	10 (76.9%)		

### Apatinib Inhibited the Proliferation and Migration of PTC Cell Lines

**Figure 2:**
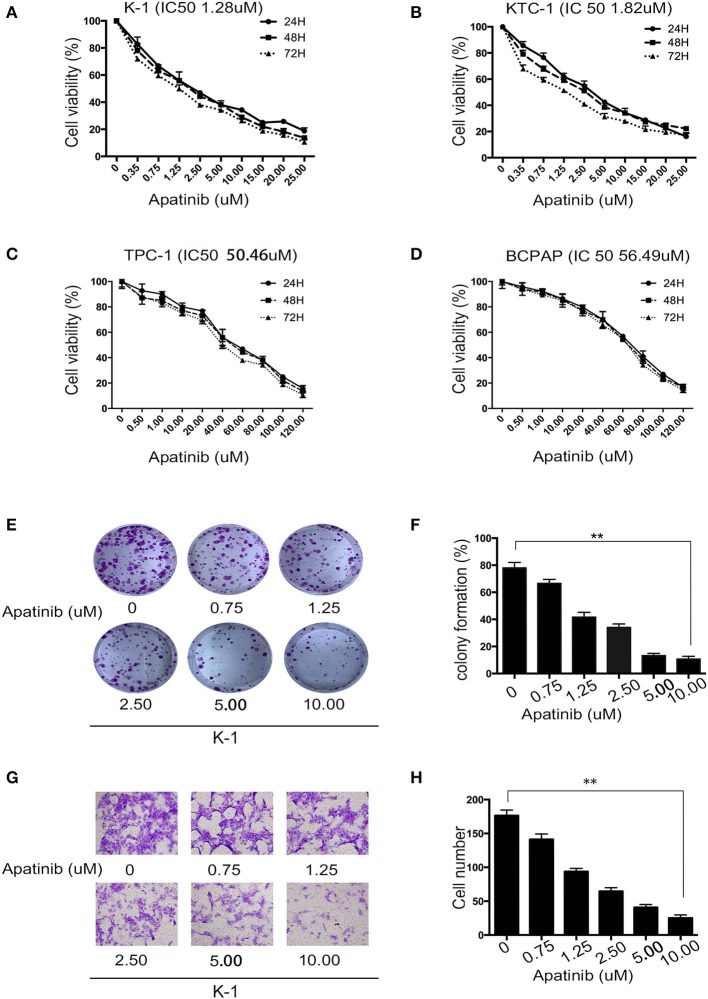
Apatinib inhibits the proliferation and migration of PTC cell lines. **(A–D)** K-1, KTC-1, TPC-1, and BCPAP cells were incubated with apatinib at various concentrations for 24, 48, and 72 h. Then, cell viability was detected by CCK8 assay, and the IC50 values of apatinib at 24 h are shown in brackets behind the name of the cell line. **(E,F)** Apatinib inhibited the long-term proliferation of K-1 cells, as measured by colony-formation assay. **(G,H)** Apatinib suppressed the migration of K-1 cells, and the migration index was measured by Transwell assay. Each experiment was performed three times. Data are expressed as the mean ± SD (*P < 0.05, **P < 0.01, ***P < 0.001).

To assess the effects of apatinib on the growth of PTC cells, we used the K-1, KTC-1, TPC-1, and BCPAP cell lines. We treated the cell lines with apatinib at increasing concentrations for 24, 48, and 72 h and used the CCK8 assay to determine cell viability. Apatinib effectively inhibited the growth of PTC cells in a dose-dependent manner (Figures 2A–D). The IC50 values for apatinib in K-1 and KTC-1 cells, which highly expressed VEGFR2, were 1.28 and 1.82 μM, respectively, at 24 h. The IC50 value for apatinib in TPC-1 and BCPAP cells, which exhibited low VEGFR2 expression, were 50.46 and 56.49 μM at 24 h, respectively. Based on these results, we used the K-1 and KTC-1 cell lines for further investigation. To further examine the effects of apatinib on PTC cell proliferation, a colony-formation assay was performed with K-1 and KTC-1 cells. Apatinib reduced the colony-formation ability of K-1 (Figures 2E,F) and KTC-1 cells (Figures S1A,B) in a dose-dependent manner. These data confirmed that apatinib suppressed the proliferation of PTC cells *in vitro*.

To examine the effects of apatinib on the migration and invasion of PTC cells, Transwell assays were carried out with K-1 and KTC-1 cells. We found that apatinib inhibited the migration and invasion of K-1 (migration assay shown in Figures 2G,H; invasion assay shown in Figures S1E,F) and KTC-1 (migration assay shown in Figures S1C,D; invasion assay shown in Figures S1G,H) cells in a dose-dependent manner. These data suggested that apatinib inhibits PTC cell migration and invasion.

### Apatinib Induced Apoptosis and Cell Cycle Arrest in PTC Cells

**Figure 3:**
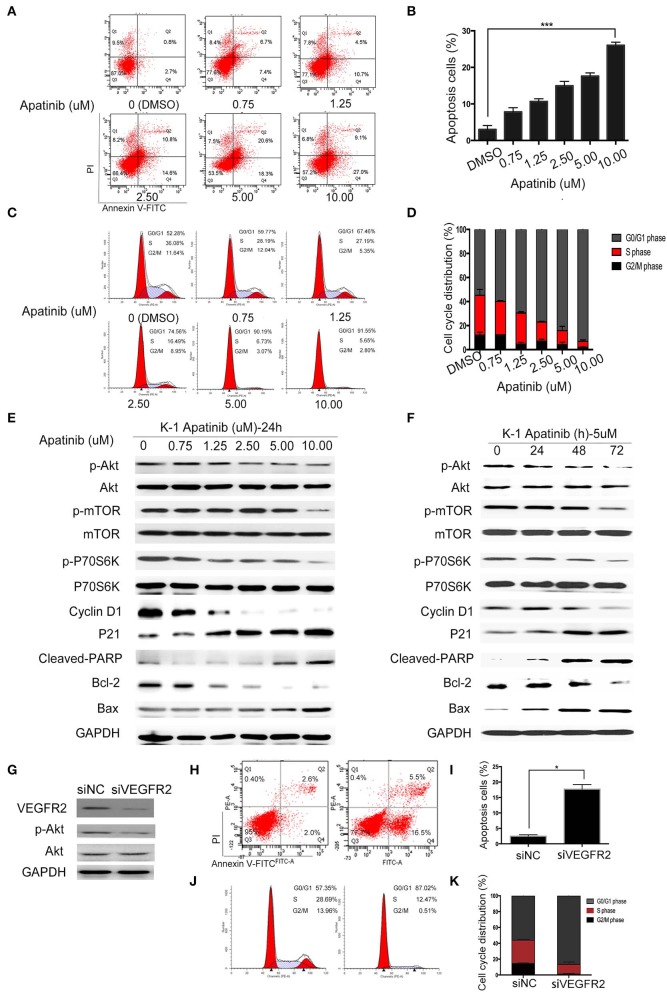
Apatinib induces apoptosis and cell cycle arrest *in vitro*. **(A,B)** Annexin V-FITC/PI staining to detect apoptosis in PTC cells induced by apatinib at various concentrations. Apoptotic cells were analyzed by flow cytometry; (AnV+) (PI–) cells were considered early apoptotic, while (AnV+) (PI+) cells were considered late apoptotic. **(C,D)** Apatinib at various concentrations caused G0/G1 arrest in PTC cells. After PI staining, the cell cycle distribution was assessed by flow cytometry. **(E,F)** The expression of proteins related to apoptosis, the cell cycle, and cell signaling pathways was determined by western blotting. **(G)** K-1 cells were transfected with VEGFR2 siRNA, the protein expression level of VEGFR2; p-Akt and Akt were detected by western blot. **(H,I)** After VEGFR2 was downregulated, the percentage of apoptotic cells were analyzed by flow cytometry; (AnV+) (PI–) cells were considered early apoptotic, while (AnV+) (PI+) cells were considered late apoptotic. **(J,K)** After VEGFR2 was downregulated, the cell cycle distribution was assessed by flow cytometry. Data are expressed as the mean ± SD (*P < 0.05, **P < 0.01, ***P < 0.001).

To confirm the effect of apatinib on PTC cells, K-1 and KTC-1 cells were treated with apatinib at various concentrations for 24 h, stained with Annexin V/FITC and PI, and analyzed by flow cytometry. The results confirmed that apatinib induced apoptosis in both K-1 (Figures 3A,B) and KTC-1 (Figures S2A,B) cells in a concentration-dependent manner. Moreover, PTC cells treated with apatinib at various concentrations for 24 h in G0/G1 phase of the cell cycle accumulated, as shown in Figures 3C,D and Figures S2C,D. To ascertain the mechanism of this effect, we examined the expression of proteins related to cell signaling (Akt, mTOR, and P70S6K), the cell cycle (cyclin D1 and P21), and apoptosis signaling (cleaved PARP, Bax, and Bcl-2) by WB. Apatinib decreased cyclin D1 expression and increased P21 expression in a dose- and time-dependent manner. Meanwhile, apatinib upregulated cleaved PARP and Bax levels and downregulated Bcl-2 levels in a dose- and time-dependent manner. Apatinib also downregulated p-Akt, p-mTOR, and p-P70S6K levels, shown in Figures 3E,F and Figures S3A–C. To verify whether the antitumor effect of apatinib is governed by the VEGFR2-mediated pathways, we knocked down in K-1 cells using siRNA. K-1 cells were transfected with VEGFR2 siRNA for 24 h. As shown in Figure 3G, VEGFR2 siRNA inhibited the expression of VEGFR2 and p-Akt. Compared with the control group, VEGFR2 downregulated group-induced apoptosis and cell cycle arrest, shown in Figures 3H–K. Therefore, all the data confirmed that apatinib can regulate the PI3K/Akt/mTOR signaling pathway and induce apoptosis and cell cycle arrest in PTC cells.

### Apatinib Induced Autophagy in PTC Cell Lines via the Akt/mTOR Signaling Pathway

**Figure 4:**
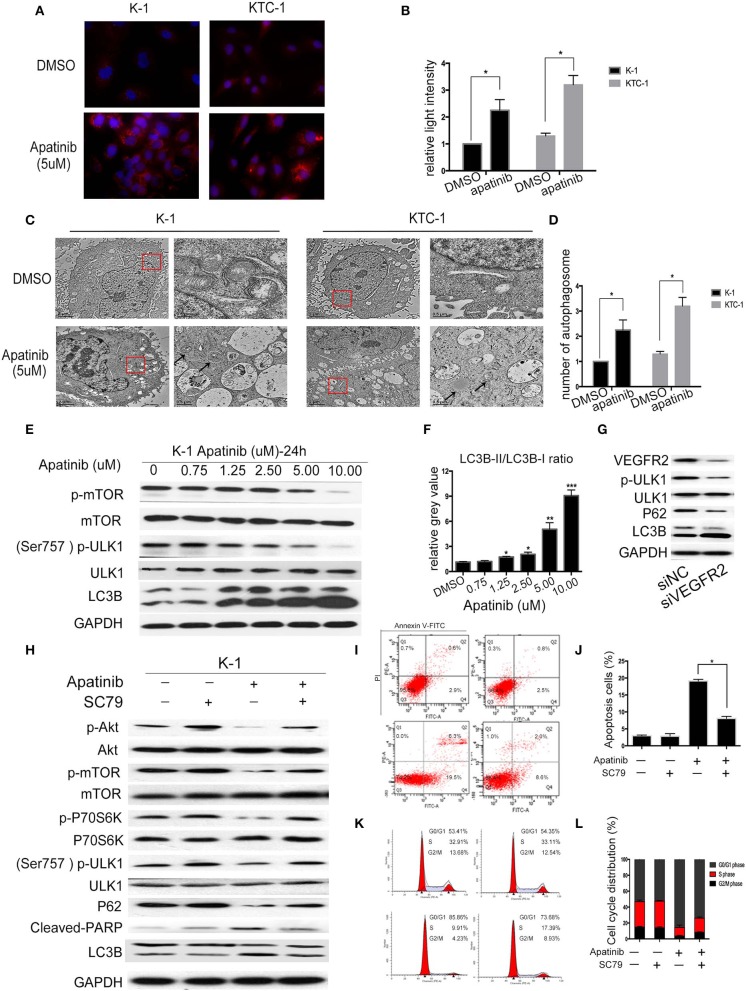
Apatinib induces autophagy in PTC cell lines via the Akt/mTOR signaling pathway. **(A,B)** Immunofluorescence images of K-1 and KTC-1 cells treated with or without 5 μM apatinib for 24 h. Original images were taken at 400× magnification. There were more LC3B puncta in the apatinib-treated group than in the control group. **(C,D)** Transition electron microscopy images of K-1 and KTC-1 cells treated with or without apatinib for 24 h. More autophagic vacuoles (AVs) were observed in the apatinib-treated group than in the control group. Arrows: autophagosomes. **(E)** The expression of proteins related to cell signaling pathways and autophagy. **(F)** The relative gray value of LC3B-II/LC3B-I. **(G)** K-1 cells were transfected with VEGFR2 siRNA, the protein expression level of VEGFR2; p-ULK1, ULK1, P62, and LC3B were detected by western blot. **(H)** K-1 cells were treated with apatinib with or without SC79 for 24 h. The expression of proteins related to apoptosis, the cell cycle, and cell signaling pathways was determined by western blotting. **(I,J)** K-1 cells were treated as **(H)**; the percentage of apoptotic cells was analyzed by flow cytometry; (AnV+) (PI–) cells were considered early apoptotic, while (AnV+) (PI+) cells were considered late apoptotic. **(K,L)** K-1 cells were treated as **(H)**; the cell cycle distribution was assessed by flow cytometry. Data are expressed as the mean ± SD (*P < 0.05, **P < 0.01, ***P < 0.001).

Autophagy is a double-edged sword in tumors, as it can cause tumor survival or death. A number of studies have found that the mechanism of TKI resistance is closely related to autophagy. Therefore, we next examined whether apatinib causes autophagy in PTC. First, we measured the expression of LC3B, a key marker in the initial stage of autophagy, using an IF assay. As shown in Figures 4A,B, apatinib-treated cells showed LC3B accumulation. Furthermore, we used transmission electron microscopy to assess the formation of autophagic vacuoles (autophagosomes). As shown in Figures 4C,D, the formation of autophagosomes in apatinib-treated cells was dramatically increased compared with that in untreated cells. Finally, we performed WB to measure protein expression, and as shown in Figures 4E,F, after treatment with apatinib at various concentrations, the expression of p-mTOR and p-ULK1 decreased, while that of LC3B increased, further confirming that apatinib induces autophagy in PTC cells.

To explore whether apatinib-induced autophagy is governed by the VEGFR2-mediated pathways, we knocked down in K-1 cells using siRNA. K-1 cells were transfected with VEGFR2 siRNA for 24 h. As shown in Figure 4G, after VEGFR2 was downregulated, the expression of VEGFR2, p-ULK1, and P62 were decreased, and the expression of LC3B was increased. The data suggested that apatinib-induced autophagy was governed by VEGFR2-mediated pathways. Many recent reports (18, 19) have indicated that the PI3K/Akt/mTOR signaling pathway negatively regulates autophagy and apoptosis in many tumors. As shown in Figure 3, apatinib could regulate the Akt/mTOR signaling pathway and induce apoptosis. To further investigate whether the Akt/mTOR signaling pathway is involved in apatinib-induced autophagy in PTC cells, we treated K-1 cells with apatinib and SC79, an agonist of AKT. As shown in Figure 4H, the expression of p-Akt, p-mTOR, p-P70S6K, p-ULK1, and P62 was increased in cells treated with both SC79 and apatinib compared with apatinib-treated cells, and the expression of cleaved PARP and LC3B was decreased, suggesting that apoptosis and autophagy were inhibited. Meanwhile, flow cytometry data showed that cells treated with both apatinib and SC79 have a lower apoptotic rate and a lower cell cycle arrest rate than cells treated with the apatinib signal group, shown in Figures 4I–L. Thus, activation of the Akt pathway could rescue the apoptosis and autophagy induced by apatinib. Taken together, these results confirmed that apatinib induces apoptosis and autophagy in PTC cells via the PI3K/Akt/mTOR signaling pathway.

### Inhibition of Autophagy Sensitized PTC Cells to Apatinib

**Figure 5:**
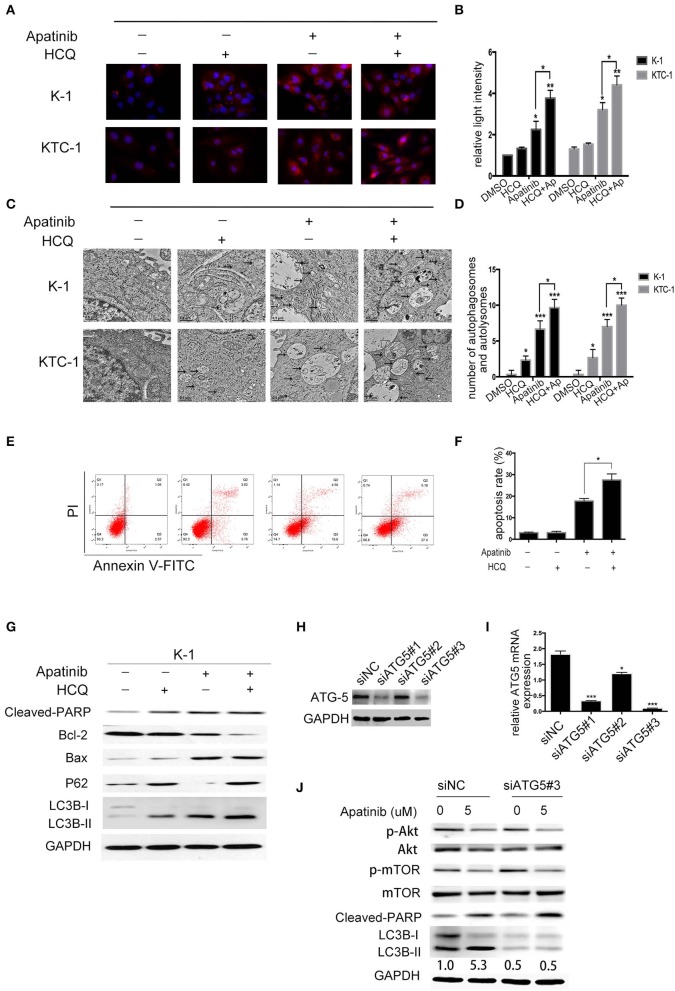
Inhibition of autophagy enhances apatinib-induced apoptosis in PTC cells. **(A, B)** Representative immunofluorescence images of K-1 and KTC-1 cells treated with DMSO, HCQ, apatinib, or a combination of apatinib and HCQ. **(C, D)** Representative transition electron microscopy images of K-1 and KTC-1 cells treated with DMSO, HCQ, apatinib, or a combination of apatinib and HCQ. Arrows: autophagosomes and autolysosomes. **(E, F)** Inhibition of autophagy with HCQ enhanced the apoptosis of apatinib-treated PTC cells. **(G)** The expression levels of autophagy- and apoptosis-related proteins were detected by western blotting after the treatment of K-1 cells with apatinib with or without HCQ. **(H)** K-1 cells were transfected with ATG5 siRNA, the protein expression levels of ATG5. **(I)** K-1 cells were transfected with ATG5 siRNA, the mRNA expression levels of ATG5. **(J)** After ATG5 downregulation, the expression levels of p-Akt, p-mTOR, cleaved PARP, and LC3B in K-1 cells with or without apatinib treatment were determined by western blotting. Data are expressed as the mean ± SD (*P < 0.05, **P < 0.01, ***P < 0.001).

To determine whether LC3B accumulation induced by apatinib is due to inhibited autophagosome degradation or enhanced autophagosome formation, we treated K-1 cells with DMSO or apatinib with/without HCQ. HCQ inhibits the final stage of autophagy, preventing the degradation of autophagosomes by increasing lysosomal pH. As shown in Figures 5A,B, among both K-1 and KTC-1 cells, LC3B expression was decreased in the cotreated group compared with the apatinib-treated group. Similarly, among K-1 and KTC-1 cells, compared with the apatinib-treated groups, the cotreated groups showed increased autophagic vacuole formation (Figures 5C,D). These data suggested that autophagy was inhibited by HCQ treatment.

To explore the role of apatinib-induced autophagy in PTC cells, we treated K-1 cells with DMSO, apatinib, HCQ, or both apatinib and HCQ and found that combination treatment was the most effective at inducing apoptosis, as evidenced by the significantly higher ratio of apoptotic cells than that in the group treated with apatinib alone (Figures 5E,F), and the same result was observed in the KTC-1 cell line (Figures S4A,B). In apatinib-treated cells, LC3B-II expression increased, and the expression of P62, a substrate of autophagosome degradation, decreased. In the cotreatment group, LC3B-II and P62 expression levels were further increased; as shown in Figure 5G, autophagy was inhibited in the cotreated group, which exhibited increased cleaved PARP and Bax expression levels and decreased Bcl-2 expression levels. Taken together, these results suggested that apatinib-induced autophagy was cytoprotective and that inhibiting autophagy could increase apoptosis.

To further determine whether HCQ functions by inhibiting autophagy rather than through an unknown mechanism, we inhibited autophagy using genetic tools. ATG5, a key gene in the formation of autophagic vacuoles, was knocked down in K-1 cells using siRNA. K-1 cells were transfected with ATG5 siRNA for 24 h and then treated with or without apatinib for another 24 h. As shown in Figures 5H–J, ATG5 siRNA inhibited the expression of ATG5 and LC3B, suggesting that autophagy had been inhibited. Compared with the group treated with negative control siRNA, the group treated with ATG5 siRNA and the apatinib combination treatment group showed significant increased cleaved PARP levels. Overall, these results demonstrated that autophagy inhibitors enhance the efficacy of apatinib in PTC cells.

### Inhibition of Autophagy Enhanced the Suppression of PTC Growth Induced by Apatinib *in vivo*

**Figure 6:**
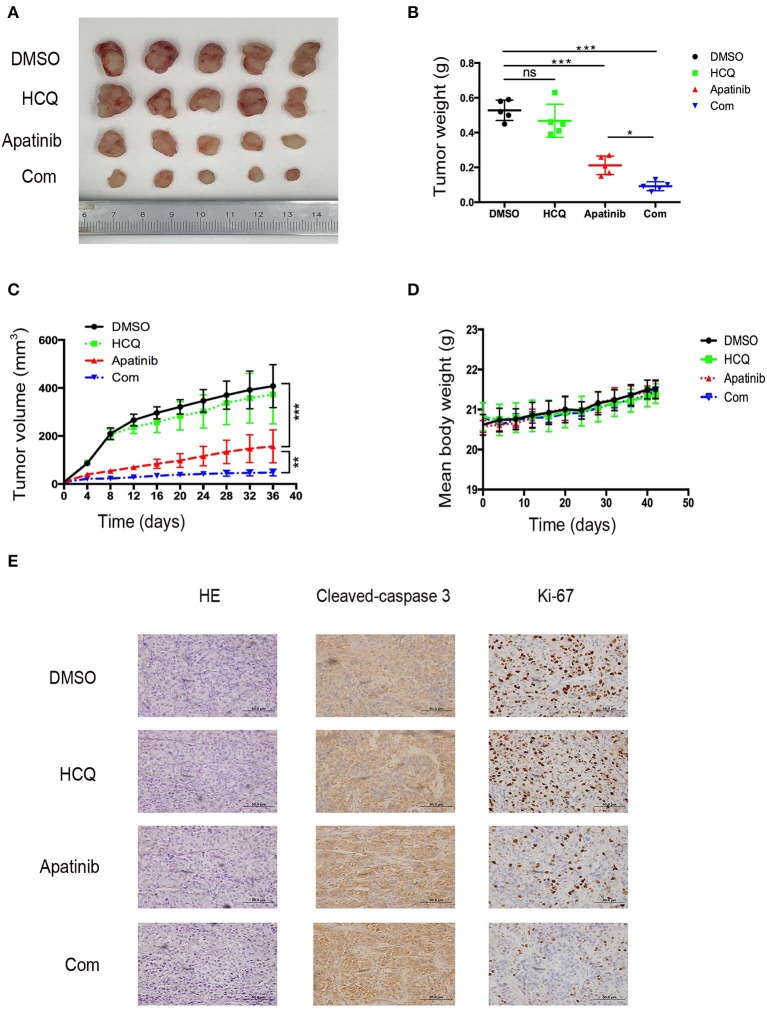
Inhibition of autophagy enhanced the suppression of PTC growth by apatinib *in vivo*. **(A)** Images of tumors dissected from mice in the four groups (DMSO control group, HCQ single-treatment group, apatinib single-treatment group, and apatinib and HCQ combined treatment group) inoculated with K-1 cells. **(B)** Tumor weights in the four groups. **(C)** Tumor volumes in the four groups. **(D)** Mouse weights in the four groups. **(E)** HE and IHC staining to detect cleaved caspase 3 and Ki-67 in the four groups. Data are expressed as the mean ± SD (*P < 0.05, **P < 0.01, ***P < 0.001).

To confirm the effect of apatinib-induced apoptosis and autophagy on PTC *in vivo*, we used K-1 cells to generate an *in vivo* xenograft model. The xenograft mice were divided into the following four groups: group A (a DMSO control group), group B (a HCQ single-treatment group), group C (an apatinib single-treatment group), and group D (an apatinib and HCQ combination treatment group). Based on our results, the DMSO control group and the HCQ single-treatment group did not show significant differences in tumor volume or tumor weight. In contrast, the apatinib single-treatment group showed significantly decreased tumor volume and tumor weight compared with the DMSO control group and the HCQ single-treatment group, and tumor volume and weight were significantly different in the HCQ combination treatment group and the apatinib single-treatment group. Moreover, Ki-67 expression in the combined treatment group was significantly decreased, and cleaved caspase3 expression was increased, implying that apoptosis was enhanced in these tumors. These results demonstrated that apatinib-mediated suppression of tumor growth and blockade of autophagy could improve the antitumor effect on PTC *in vivo*, as shown in Figure 6. Taken together, these results suggested that autophagy inhibitors enhance the efficacy of apatinib in PTC and that given their low toxicity, these inhibitors can be an effective curative therapy in PTC.

## Discussion

PTC is the most common type of thyroid cancer and has a favorable prognosis. However, a small proportion of PTC patients develop metastatic disease and are resistant to conventional therapies, including thyrotropin-suppressive therapy and radioactive iodine; the outlook in these patients is therefore dim, as current treatment options for these patients are limited. Apatinib is a novel TKI highly selective for VEGFR2 with good antitumor effects in various types of solid tumors. Apatinib was shown to be safe and highly efficacious in a small number of patients with radioiodine-refractory differentiated thyroid cancer (13). In this report, we investigated the potential therapeutic effect of apatinib on PTC and the mechanism of apatinib *in vitro* and *in vivo*.

Apatinib was reported to suppress angiogenesis in anaplastic thyroid carcinoma by blocking the Akt/GSK3/ANG pathway (20–22); however, the effect of apatinib in PTC is unclear. In this report, we discovered that apatinib could inhibit PTC cell growth and migration and the induction of apoptosis. The PI3K/Akt signaling pathway plays an important role in a variety of tumors, including thyroid cancer, and is closely related to tumorigenesis, proliferation, invasion, apoptosis, and autophagy. Our data showed that p-Akt levels were decreased after apatinib treatment in a dose- and time-dependent manner.

Thus, we suggest that apatinib promotes apoptosis through the PI3K/Akt/mTOR signaling pathway. Autophagy is a ubiquitous cellular mechanism for the maintenance of homeostasis. In tumor cells, autophagy plays a dual role as it can promote death or survival. Some studies have confirmed that TKI resistance is associated with increased autophagic activity (23–25). In this report, we found that apatinib induced autophagy in PTC, as it does in other types of tumors. Moreover, we used HCQ, an autophagy inhibitor, in combination with apatinib to inhibit autophagic flux and increase apoptosis, further confirming that apatinib-induced autophagy is a protective mechanism in tumor cells. Moreover, the same results were obtained in ATG5-knockdown PTC cells, and these results were further confirmed at the genetic level. The Akt signaling pathway closely interacts with mTOR to influence autophagy, and in this study, we found that after apatinib treatment, the levels of p-mTOR, p-P70S6K, and p-ULK1 were decreased. Moreover, apatinib cotreatment with SC79, an activator of Akt, reduced apoptosis and autophagy. Thus, we demonstrated that apatinib can induce apoptosis and autophagy via the PI3K/Akt/mTOR signaling pathway and that this pathway might be a novel mechanism of drug resistance. Furthermore, the blockade of autophagy could enhance the sensitivity of PTC to apatinib.

In summary, our data show that apatinib exerted antitumor effects in PTC by suppressing tumor growth, promoting apoptosis, and inhibiting migration through the PI3K/Akt/mTOR signaling pathway. Additionally, apatinib could induce autophagy. Importantly, apatinib-induced autophagy was cytoprotective in PTC cells, and the blockade autophagy could improve apoptosis *in vitro* and *in vivo*. Thus, these findings reinforce our research findings and the clinical application of apatinib and indicate that autophagy is a new molecular therapeutic target in PTC. Combination treatment with apatinib and an autophagy inhibitor may be a useful therapeutic strategy for refractory PTC, and our data provide support for future clinical trials of autophagy inhibitors.

## Data Availability Statement

All datasets generated for this study are included in the article/Supplementary Material.

## Ethics Statement

The studies involving human participants were reviewed and approved by Ethics Committee of Tianjin Cancer Institute and Hospital. The patients/participants provided their written informed consent to participate in this study. The animal study was reviewed and approved by Ethics Committee of Tianjin Cancer Institute and Hospital. Written informed consent was obtained from the individual(s) for the publication of any potentially identifiable images or data included in this article.

## Author Contributions

Conceptualization: XZ. Data curation and investigation: XM and HW. Formal analysis: JZhao and LH. Funding acquisition: XZ, JZhi, SW, XR, DL, YZ, ZM, WY, and LG. Methodology: BQ and MG. Project administration: HZ and MG. Resources: XZ and MG. Software: XH, JZhang, YW, HZ, XZ, and MG. Writing—original draft and writing—review and editing: XM.

### Conflict of Interest

The authors declare that the research was conducted in the absence of any commercial or financial relationships that could be construed as a potential conflict of interest.

## Abbreviations

PTC: papillary thyroid carcinoma
VEGFR2: vascular endothelial growth factor receptor-2
ATG5: autophagy-associated gene 5
TKIs: tyrosine kinase inhibitors.
