# Supplementary figures and images for: Apatinib Inhibits Cell Proliferation and Induces Autophagy in Human Papillary Thyroid Carcinoma via the PI3K/Akt/mTOR Signaling Pathway
Source: Front Oncol. 2020 Mar 11;10:217. doi: 10.3389/fonc.2020.00217 (PMC7078169; doi:10.3389/fonc.2020.00217)

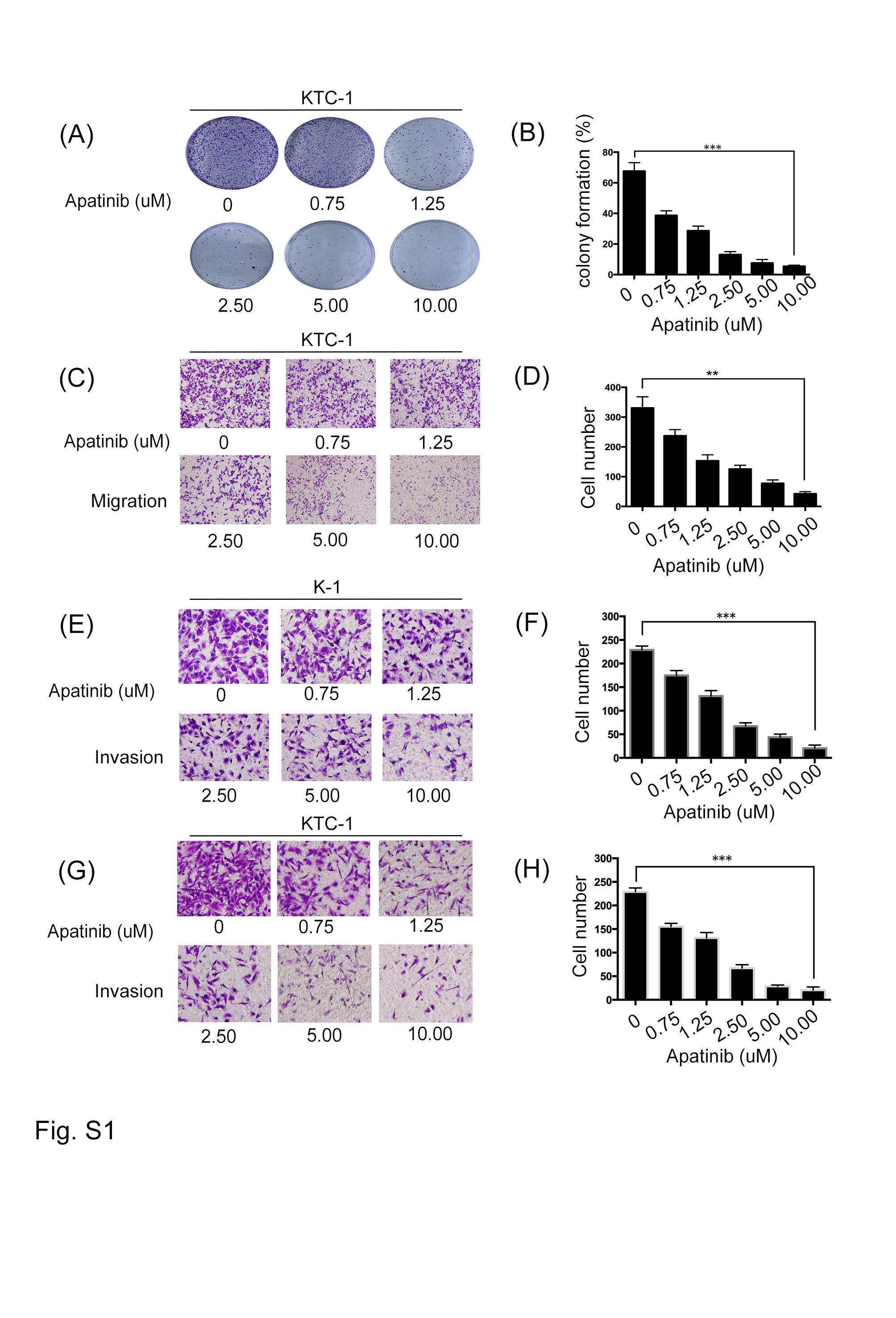

Supplement: Supplementary file 1 [file Image_1.tif]

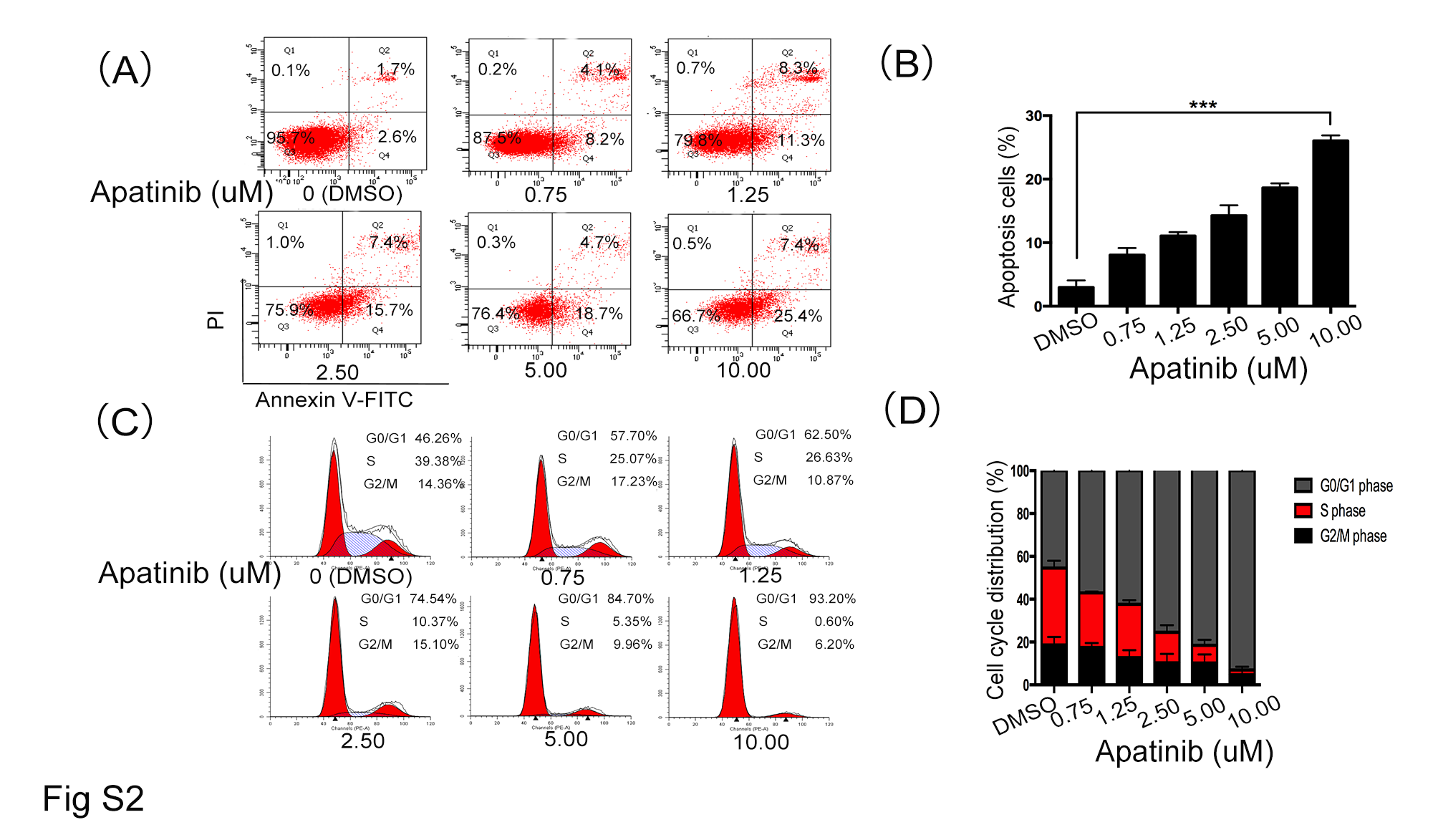

Supplement: Supplementary file 2 [file Image_2.TIF]

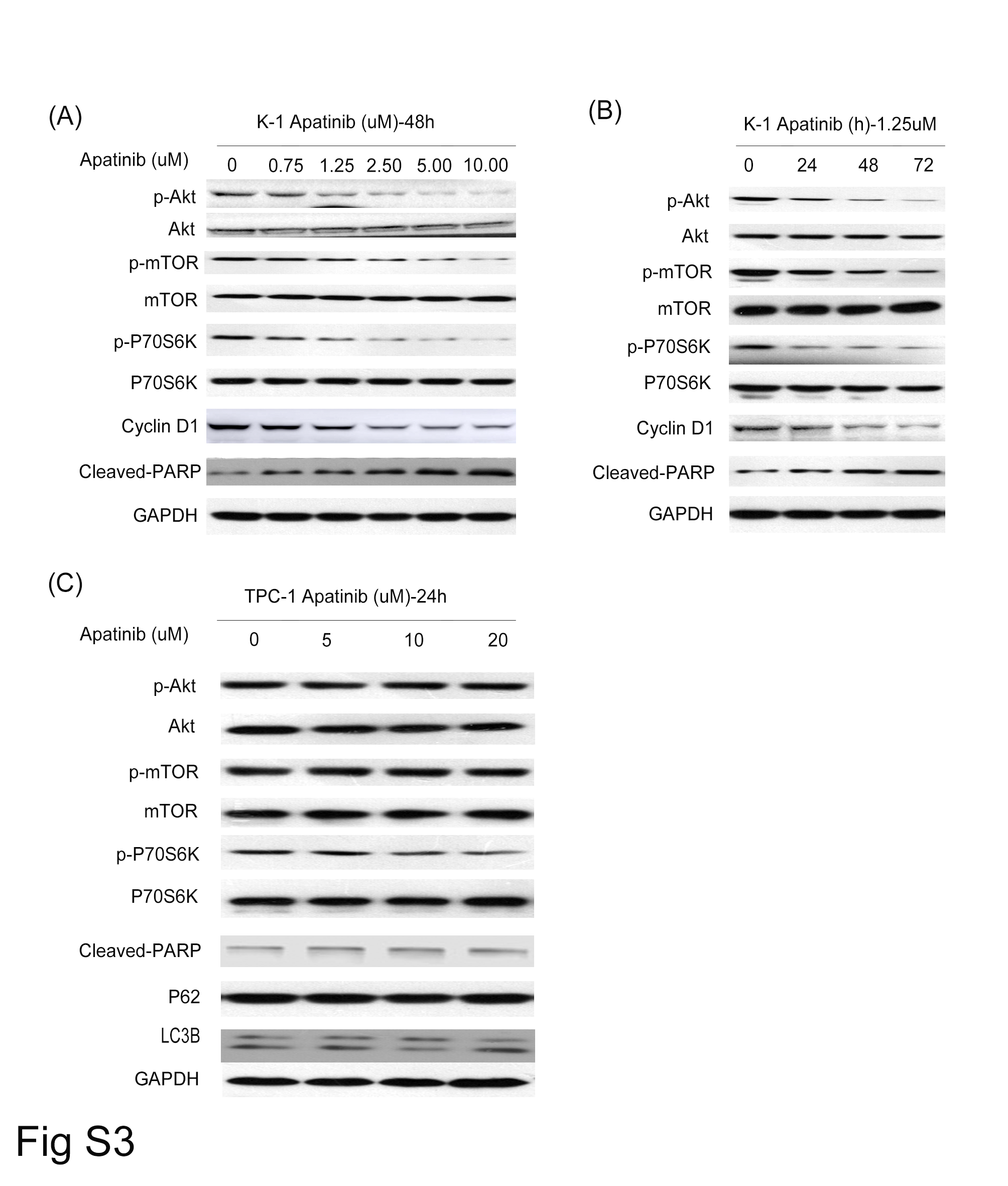

Supplement: Supplementary file 3 [file Image_3.tif]

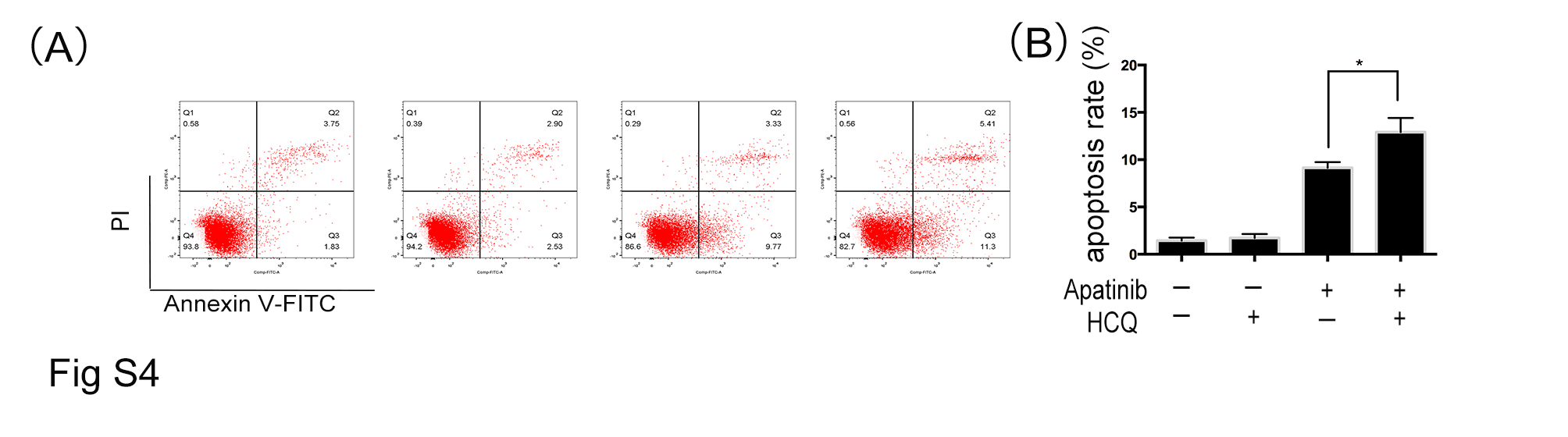

Supplement: Supplementary file 4 [file Image_4.tif]
