# Supplementary material for: Apatinib Inhibits Cell Proliferation and Induces Autophagy in Human Papillary Thyroid Carcinoma via the PI3K/Akt/mTOR Signaling Pathway
Source: Front Oncol. 2020 Mar 11;10:217. doi: 10.3389/fonc.2020.00217 (PMC7078169; doi:10.3389/fonc.2020.00217)
Supplement: Supplementary file 5 [file Table_1.DOCX]

**Fig. S1.** Apatinib inhibits the proliferation , migration and invasion of PTC cells

(A) and (B) Apatinib inhibited the long-term proliferation of KTC-1 cells as measured by colony formation assay. (C) and (D) Apatinib suppressed the migration of KTC-1 cells, and the migration index was measured by transwell assay. (E) and (F) Apatinib suppressed the invasion of K-1 cells, and the invasion index was measured by transwell assay. (G) and (H) Apatinib suppressed the invasion of KTC-1 cells, and the invasion index was measured by transwell assay.

Each experiment was performed three times.

**Fig. S2.** Apatinib induces apoptosis and cell cycle arrest in KTC-1 cells (A) and (B) Annexin V-FITC/PI staining of apoptosis induced by various concentrations of apatinib in KTC-1 cells. Apoptotic cells were analyzed by flow cytometry. (C) and (D) Various concentrations of apatinib caused G0/G1 arrest in KTC-1 cells. After PI staining, the cell cycle distribution was assessed by flow cytometry. Each experiment was performed three times.

**Fig. S3.** Apatinib induced apoptosis and autophagy in K-1 cells

(A) K-1 cells were incubated with apatinib at various concentrations for 48h, the expression of proteins related to apoptosis, the cell cycle, and cell signaling pathways was detected by western blotting. (B) K-1 cells were incubated with 1.25 uM apatinib for 24h, 48h, 72h, he expression of proteins related to apoptosis, the cell cycle, and cell signaling pathways was detected by western blotting. (C) TPC-1 cells, which exhibited low VEGFR2 expression, were incubated with apatinib at various concentrations for 24h, the expression of proteins related to apoptosis, autophagy and cell signaling pathways was detected by western blotting.

**Fig. S4.** Inhibition of autophagy enhances apatinib-induced apoptosis in KTC-1 cells

(A) Inhibition of autophagy with HCQ enhanced the apoptosis of apatinib-treated PTC cells. (B) The apoptosis rate of the cells was detected by Fig (A). Each experiment was performed three times.
